# The Risk of Spontaneous Abortion Does Not Increase Following First Trimester mRNA COVID-19 Vaccination

**DOI:** 10.3390/jcm11061698

**Published:** 2022-03-18

**Authors:** Ioana Mihaela Citu, Cosmin Citu, Florin Gorun, Ioan Sas, Felix Bratosin, Andrei Motoc, Bogdan Burlea, Ovidiu Rosca, Daniel Malita, Oana Maria Gorun

**Affiliations:** 1 Department of Internal Medicine I, Victor Babes University of Medicine and Pharmacy Timisoara, Eftimie Murgu Square 2, 300041 Timisoara, Romania; citu.ioana@umft.ro; 2 Department of Obstetrics and Gynecology, Victor Babes University of Medicine and Pharmacy Timisoara, Eftimie Murgu Square 2, 300041 Timisoara, Romania; gorun.florin@umft.ro (F.G.); sasioan56@yahoo.com (I.S.); 3 Methodological and Infectious Diseases Research Center, Department of Infectious Diseases, Victor Babes University of Medicine and Pharmacy, Eftimie Murgu Square 2, 300041 Timisoara, Romania; felix.bratosin7@gmail.com (F.B.); ovidiu.rosca@umft.ro (O.R.); 4 Department of Anatomy and Embryology, Victor Babes University of Medicine and Pharmacy Timisoara, Eftimie Murgu Square 2, 300041 Timisoara, Romania; amotoc@umft.ro; 5 Department of Obstetrics and Gynecology, Municipal Emergency Clinical Hospital Timisoara, 1-3 Alexandru Odobescu Street, 300202 Timisoara, Romania; bogdanburlea@yahoo.com (B.B.); oanabalan@hotmail.com (O.M.G.); 6 Department of Radiology, Victor Babes University of Medicine and Pharmacy Timisoara, Eftimie Murgu Square 2, 300041 Timisoara, Romania; malita.daniel@umft.ro

**Keywords:** SARS-CoV-2, COVID-19, pregnancy vaccination, first trimester

## Abstract

Clinical trials for COVID-19 vaccines initially excluded pregnant women due to safety concerns, and when the vaccines were authorized for emergency use, they were not recommended for this population. However, observational studies discovered that pregnant women infected with COVID-19 have higher risks of negative pregnancy and delivery outcomes compared to non-pregnant women, raising the question of the risks–benefits of administering COVID-19 vaccines to pregnant women. By mid-2021, there was general consensus on the relative safety of COVID-19 vaccination during pregnancy; therefore, it is critical to investigate the safety issues related to these vaccines, considering the increasing acceptance among pregnant women. To address these concerns, we developed a research project to study the short-term effects and outcomes of COVID-19 vaccination during the first trimester of pregnancy. Our research followed an observational retrospective design for 12 months from the beginning of the vaccination campaign, and included 124 cases of spontaneous abortions and 927 ongoing pregnancies. The odds of spontaneous abortion were non-significant for both versions of the mRNA vaccine (Pfizer BNT162b2 AOR = 1.04, CI = 0.91–1.12; Moderna mRNA-1273 AOR = 1.02, CI = 0.89–1.08). Overall, our data indicated that the risk of spontaneous abortion after mRNA COVID-19 immunization during the first trimester of pregnancy is commensurate with the predicted risk in non-vaccinated pregnant women. These findings contribute to the growing body of information regarding the safety of mRNA COVID-19 vaccination during pregnancy.

## 1. Introduction

Vaccine development in the European Union (EU) begins with preclinical in vitro and in vivo testing in the laboratory, followed by clinical testing in phase 1 conducted on healthy volunteers [1]. After confirming their safety and pharmacokinetics in humans, vaccines are progressed to phase 2 trials, which continue to assess their safety and pharmacokinetics in the targeted patient population and typically establish the dose to be used in phase 3 trials, which are larger, pivotal clinical trials that evaluate the vaccine’s efficacy [2]. In essence, these clinical studies collect detailed information about how vaccinations function and guarantee that their benefits surpass any possible adverse effects or hazards [3]. Once sufficient proof has been acquired via research and clinical trials, the companies creating the vaccines may submit a marketing authorization application to the European Medicines Agency [4].

During pregnancy, many dynamic changes occur to prevent adverse immune reactions to the fetus and to protect both the mother and fetus from infections, but it is still unclear how these immune responses contribute to changes in infection risk [5,6,7,8]. It was hypothesized that the physiological immune modulation that occurs during pregnancy might make pregnant females more susceptible to viral infections with possible, in certain circumstances, serious consequences such as embryo/fetus loss [9]. Another pregnancy-associated physiological change is the decreased functional residual lung capacity that must be addressed as it increases the risk of severe pneumonia. This risk further increases when associated with COVID-19 [10,11,12]. Several research studies have already confirmed the increased negative outcomes of pregnancies associated with SARS-CoV-2 infection [13,14,15], although it seems that vertical transmission is not necessarily a contributing factor to these outcomes [16,17].

Pregnant women represent a population that was not included in the initial clinical trials for COVID-19 vaccine authorization due to safety concerns about the new mRNA vaccines [18,19]. However, it is historically proven that vaccination during pregnancy benefits both the mother and the newborn by avoiding the risk of severe disease. Pregnant women have an increased risk of morbidity, negative pregnancy outcomes and death from vaccine-preventable diseases. Influenza, tetanus, diphtheria, and pertussis are among the vaccine-preventable infectious illnesses for which regular prenatal vaccines are suggested [20,21]. Vertical transmission from mother to fetus can be prevented by avoiding the SARS-CoV-2 infection or by the passive immunity conferred on the newborn by maternal vaccine-induced transplacental antibody transfer, which can provide protection for up to six months of life [22,23].

The COVID-19 vaccines, particularly the mRNA vaccines, such as BNT162b2 Pfizer and mRNA-1273 Moderna, were authorized by the Food and Drug Administration (FDA) in late 2020 [24], and a worldwide vaccination campaign was launched in which the vaccines were delivered in two doses. Later, at the beginning of 2021, the World Health Organization endorsed the availability of COVID-19 vaccines for pregnant women [25], which triggered the same reaction in Romania, although with a delayed response in mid-2021. As the advice of gynecologists to pregnant patients is to consider getting the COVID-19 vaccines, we developed a study aiming to address their concerns. Our focus was particularly on the first trimester when most genetic mutations and fetal malformations occur. Therefore, the end-point of this research was to determine whether pregnant women vaccinated with an mRNA-type vaccine during the first trimester have higher risks of spontaneous abortion.

## 2. Materials and Methods

Our research followed an observational retrospective design to observe adult pregnant women vaccinated with the Comirnaty^®^ Pfizer/BioNTech BNT162b2 (Marburg, Germany) or the Spikevax^®^ Moderna mRNA-1273 (Cambridge, MA, USA) during their first trimester of pregnancy in comparison with unvaccinated pregnant women, between January 2020 and January 2022. Data for unvaccinated pregnant women were retrieved from the beginning of the COVID-19 pandemic in January 2020, while data for vaccinated pregnant women were collected from the beginning of the COVID-19 vaccination campaign in January 2021. All patients/pregnancies included in the study were followed in the outpatient setting of the Obstetrics and Gynecology Clinic of the Timisoara Municipal Emergency Hospital. The research protocol was approved by the Ethics Committee of the “Victor Babes” University of Medicine and Pharmacy in Timisoara, Romania, and by the Ethics Committee of the Timisoara Municipal Hospital.

All patients evaluated in the outpatient clinic of our university-affiliated hospital are required to sign an informed consent form that allows data retrieval for research purposes. Data collection consisted of a database interrogation and as search for the patient’s personal paper records by trained medical personnel. We compared the probabilities of getting a COVID-19 vaccination 4 weeks before spontaneous abortion to the odds of receiving one in the 4 weeks preceding index dates for continuing pregnancies. Data from a total of 3094 pregnant women were included in the final analysis.

The inclusion criteria comprised all pregnancies in mothers older than 18 years, that were evaluated from the start of their first trimester during the study period in our clinic. Only pregnant women vaccinated with the Pfizer BNT162b2 or Moderna mRNA-1273 were included. Patients who did not provide consent were excluded from this research. An initial evaluation considered the differences between vaccinated and unvaccinated mothers, while the second evaluation was stratified by the pregnancy outcome in vaccinated mothers only. Both spontaneous abortions and continued pregnancies were classified according to gestational age (1–6 weeks, and 7–13 weeks), maternal age (<35 years, and ≥35 years), weight as determined by body mass index (normal weight = <25 kg/m^2^/overweight = ≥25 kg/m^2^), parity status (nulliparous, primiparous, or multiparous), vaccine type (Pfizer BNT162b2 or Moderna mRNA-1273), number of doses (1, 2, or 3), existent chronic conditions affecting the mother (pregestational diabetes mellitus, cardiac disease, essential hypertension, respiratory disease, and autoimmune disease), which were represented as a dichotomous variable (yes or no), previous miscarriage (yes or no), smoking status (smoking/not smoking), karyotype analysis (normal or abnormal), assisted reproductive techniques (yes or no), abnormal uterine or cervical anatomy (yes or no), status regarding SARS-CoV-2 infection (yes or no), number of COVID-19 vaccine doses (1, 2, or 3), and other vaccines administered during the first trimester (Tdap, HPV, influenza).

The statistical analysis was performed with IBM SPSS v.26. We computed the absolute and relative frequencies that were associated with each of the studied variables. For a comparison of proportions, we used the Chi-square and Fisher’s tests. A Shapiro–Wilk test determined the normality of continuous variables, after which the Student’s *t*-test was used to compare the means. The variables found to have significant differences between comparison groups were included in a multivariate analysis adjusted for confounding factors. The significance threshold was set as an alpha value of 0.05.

## 3. Results

During the study period, we identified a total of 3094 pregnancies with a first trimester presentation in the outpatient clinic, of which 927 had received at least one dose of mRNA vaccine. There were 124 (13.4%) cases of spontaneous abortions among those who received the COVID-19 vaccine, and 271 (12.5%) spontaneous abortions identified in pregnant women who were not vaccinated against SARS-CoV-2 infection (*p*-value = 0.506) (Table 1). The average age of women who decided to vaccinate against SARS-CoV-2 during their first trimester of pregnancy was significantly lower than those who refused (29.5 years vs. 31.6 years, *p*-value < 0.001). However, in the first group, there were significantly more infections with SARS-CoV-2 before pregnancy (15.9% vs. 8.9%, *p*-value < 0.001).

**Figure 1:**
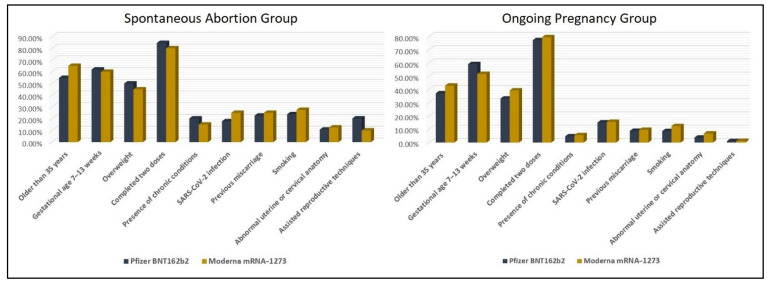
Baseline Characteristics Comparison between Women who Suffered a Spontaneous Abortion and Women Who Carried a Pregnancy until Delivery, Stratified by COVID-19 Type.

A total of 638 pregnant women were vaccinated with Pfizer BNT162b2, and 289 opted for Moderna mRNA-1273. Among the women vaccinated with at least one dose of Pfizer BNT162b2 or Moderna mRNA-1273, the proportion of women who suffered spontaneous abortions was significantly higher in women older than 35 years (58.1%), while just 39.0% of the ongoing pregnancies were women over 35 (*p*-value < 0.001). Similar findings were observed in regard to weight; 48.4% of patients with spontaneous abortions were overweight, compared with 35.1% in the ongoing pregnancies (*p*-value = 0.004). Other significant differences between the study groups were observed in the proportion of chronic conditions, previous miscarriages, number of smokers, abnormal uterine or cervical anatomy, and utilization of assisted reproductive techniques in the population with spontaneous abortions, where all the aforementioned variables had significantly higher proportions (Table 2). We did not observe important variations between the study groups in terms of SARS-CoV-2 infection status, COVID-19 vaccine type and number of doses. Similarly, there were no significant differences observed between the group of pregnant women vaccinated during the first trimester of pregnancy that had an abortion and the group who carried a pregnancy until delivery, based on the type of vaccine received (Figure 1).

**Figure 2:**
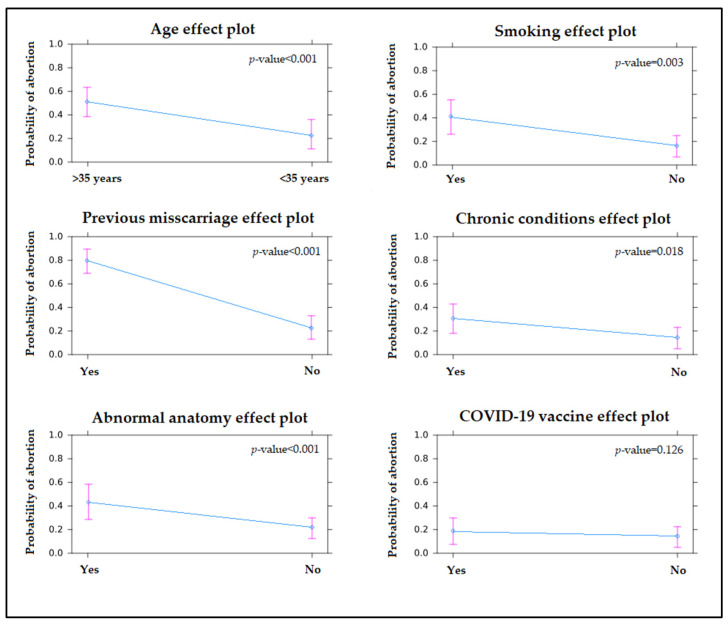
Probability of spontaneous abortion by the effect of independent risk factors.

The risk analysis, presented in Table 3, did not identify the mRNA SARS-CoV-2 vaccines, or the number of doses as increasing the odds of spontaneous abortion if administered in the third trimester of pregnancy. Similar findings were obtained for SARS-CoV-2 infection before conceiving. However, previous miscarriages and a maternal age higher than 35 years were the strongest independent risk factors for spontaneous abortion, as described in Table 3 and Figure 2.

## 4. Discussion

This article details our study of pregnant women who received an mRNA SARS-CoV-2 vaccine in the first trimester. As one of the most frequent complications of early pregnancy is the spontaneous abortion, our study observed that the number of spontaneous abortions during the first trimester in COVID-19-vaccinated pregnant women was not significantly different from the normal incidence in the first trimester in unvaccinated pregnant women, as found in the control group, where pregnancy loss in the first trimester can occur in up to 20% of pregnancies [26]. In accordance with previously reported data [27], we noticed no concerning patterns for spontaneous abortions in the first trimester. However, other independent variables such as smoking, abnormal reproductive system anatomy, history of miscarriage, age and existing comorbidities were identified as significant risk factors in this study. These findings are consistent with previous data regarding spontaneous abortion determinants [28]. Therefore, this study provides a significant contribution to the existing body of literature with findings related to a specific and relatively homogenous population of Romanian pregnant women. Our results should contribute to increasing the trust and acceptability of COVID-19 vaccines among pregnant women, considering the reluctance observed in this population [29]. Furthermore, these findings are important for inclusion in a meta-analysis regarding COVID-19 vaccination during pregnancy.

A bigger study that took place in the US reviewed the safety of mRNA vaccination against SARS-CoV-2 during all trimesters of pregnancy, or just before conception [30]. They reported that the distribution of negative pregnancy and birth complications such as fetal loss, preterm birth, small size for gestational age, congenital abnormalities, and stillbirths among participants with completed pregnancies was comparable to previously published rates in pregnant populations studied prior to the COVID-19 pandemic [31,32,33]. The largest study to date included a review of almost fifty thousand pregnant women who received mRNA vaccines for COVID-19. The available results indicated that mRNA-based vaccines (Pfizer-BioNTech and Moderna), which were also evaluated in our study, may be effective in preventing future SARS-CoV-2 infection. These immunizations have not been shown to cause obvious damage during pregnancy, excepting short-term side effects such as pain at the injection site, weariness, and headache. Antibody responses were fast after the first dosage of the vaccination, and the antibody responses were improved after the booster and were related with greater transplacental antibody transfer. Increased antibody fetal IgG and a superior antibody transfer ratio were also associated with longer intervals between the first vaccine dose and delivery. In agreement with our results, other large reviews and cohort studies did not identify any significant associations of COVID-19 vaccines with increased numbers of spontaneous abortions [34,35,36,37].

Despite the potentially fatal effects of SARS-CoV-2 infection in pregnant women and the availability of safe and effective COVID-19 immunization in non-pregnant populations, there is a lack of published evidence addressing the safety or effectiveness of any COVID-19 vaccine in human pregnancy. The approval of vaccines for use in pregnant or lactating women is based on a rigorous evaluation of observational data, clinical case reports, and clinical trials. Recognizing the critical need for pregnant women to be included in COVID-19 vaccine clinical trials, the Food and Drug Administration (FDA) recommended that developmental and reproductive toxicology studies (DART) be conducted prior to enrolling pregnant women or individuals who are not actively avoiding pregnancy in clinical trials. The developmental and reproductive toxicology studies are meant to determine the impact of new medications or vaccinations on the whole reproductive range in animals. On 4 December 2020, Moderna, whose vaccine is based on mRNA, submitted these data to the FDA [38]. Pfizer has launched a worldwide phase 2/3 study to assess the COVID-19 vaccine’s safety, tolerability, and immunogenicity in pregnant women [39]. The research will be a randomized, placebo-controlled, observer-blind study including 4000 healthy pregnant women who will get the vaccine between 24 and 34 weeks of gestation [40]. Moderna is also developing a prospective observational study and a registry to evaluate obstetric, neonatal, and newborn outcomes. Additionally, the Johnson & Johnson company (New Brunswick, NJ, USA), is preparing a phase 2 placebo-controlled study with more than 800 pregnant females.

Although our study is important and it has a sizeable cohort of patients and the existence of a control group of unvaccinated pregnant women for statistical analysis, several limitations, including the homogeneity of the participants in terms of ethnic origin, background and occupation, and the retrospectively-collected data, could bias the selection, classification and results. Another possible limitation worth noting is the lack of information regarding the outcome of pregnancies in terms of congenital malformations and stillbirths in the cohort of patients who received the COVID-19 vaccine in the first trimester. We also believe that future studies should report symptoms that occurred after vaccination in the first trimester, and other short-term complications that were not quantified in our research.

## 5. Conclusions

This study determined that the risk of spontaneous abortion after mRNA COVID-19 immunization during the first trimester of pregnancy is similar to the risk in non-vaccinated pregnant women. These findings contribute to the growing body of information regarding the safety of mRNA COVID-19 vaccination during pregnancy.

## Figures and Tables

**Table 1:** Comparison of pregnancies in the first trimester by mRNA COVID-19 vaccination status.

Variables *	Total (*n* = 3094)	Vaccine (*n* = 927)	No Vaccine (*n* = 2167)	*p*-Value
Age, years (mean ± SD)	30.7 ± 6.1	29.5 ± 7.3	31.6 ± 5.6	<0.001 ^t^
Weight, BMI (mean ± SD)	23.9 ± 5.4	24.1 ± 5.2	23.8 ± 5.4	0.152 ^t^
Previous SARS-CoV-2 infection	341 (11.0%)	148 (15.9%)	193 (8.9%)	<0.001
Nulliparous	1973 (63.8%)	584 (62.9%)	1389 (64.1%)	0.560
Infertility treatment	106 (3.4%)	30 (3.2%)	76 (3.5%)	0.704
Spontaneous abortion	395 (12.8%)	124 (13.4%)	271 (12.5%)	0.506
Abnormal karyotype	30 (0.9%)	11 (1.2%)	19 (0.9%)	0.420
Pregnancy complications **	229 (7.4%)	64 (6.9%)	165 (7.6%)	0.489
Chronic conditions ***	199 (6.4%)	63 (6.8%)	136 (6.3%)	0.589
Frequent smoking	394 (12.7%)	110 (11.9%)	284 (13.1%)	0.343

* Data reported as n(frequency) unless specified differently; ** Pregnancy complications include: high fever, hyperemesis gravidarum, vaginal bleeding, abdominopelvic pain, ectopic pregnancy, and gestational trophoblastic disease; ^t^—Unpaired Student’s *t*-test. *** Chronic conditions include pregestational diabetes mellitus, cardiac disease, essential hypertension, respiratory disease, and autoimmune disease.

**Table 2:** Characteristics of first trimester pregnant women who received at least one dose of mRNA COVID-19 vaccine.

Characteristics *	Spontaneous Abortion (*n* = 124)	Ongoing Pregnancy (*n* = 803)	*p*-Value
**Age**			<0.001
<35	52 (41.9%)	490 (61.0%)	
≥35	72 (58.1%)	313 (39.0%)	
Weight **			0.004
Normal (<25 kg/m^2^)	64 (51.6%)	521 (64.9%)	
Overweight (≥25 kg/m^2^)	60 (48.4%)	282 (35.1%)	
**Gestational age**			0.358
1–6 weeks	48 (38.7%)	346 (43.1%)	
7–13 weeks	76 (61.3%)	457 (56.9%)	
**Vaccine**			0.779
Pfizer BNT162b2	84 (67.7%)	554 (68.9%)	
Moderna mRNA-1273	40 (32.3%)	249 (31.1%)	
**Number of doses**			0.232
1	17 (13.7%)	120 (14.9%)	
2	103 (83.1%)	626 (77.9%)	
3	4 (3.2%)	57 (7.2%)	
**Other vaccines given during 1st trimester**			
Tdap	83 (66.9%)	519 (64.6%)	0.619
HPV	7 (5.6%)	62 (7.7%)	0.412
Influenza	42 (33.9%)	271 (33.7%)	0.978
Chronic conditions ***			<0.001
Yes	23 (18.5%)	40 (4.9%)	
No	101 (81.5%)	763 (95.1%)	
**SARS-CoV-2 infection**			0.175
Yes	25 (20.1%)	123 (15.3%)	
No	99 (79.9%)	680 (84.7%)	
**Previous miscarriage**			<0.001
Yes	29 (23.3%)	73 (9.1%)	
No	95 (76.7%)	730 (90.9%)	
**Smoking status**			<0.001
Smoker	31 (25.0%)	79 (9.8%)	
Non-smoker	93 (75.0%)	724 (90.2%)	
**Abnormal uterine or cervical anatomy**			0.003
Yes	14 (11.3%)	38 (4.7%)	
No	110 (88.7%)	765 (95.3%)	
**Karyotype analysis (*n* = 37)**			
Normal	26 (70.3%)	-	
Abnormal	11 (29.7%)	-	
**Assisted reproductive techniques**			<0.001
Yes	21 (16.9%)	9 (1.1%)	
No	103 (83.1%)	794 (98.9%)	

* Data reported as n (frequency) unless specified differently; ** Weight adjusted for gestational age; *** Chronic conditions include pregestational diabetes mellitus, cardiac disease, essential hypertension, respiratory disease, and autoimmune disease; karyotype analysis was performed by request to determine if the reason for spontaneous abortion was a genetic anomaly.

**Table 3:** Adjusted odds ratios for a spontaneous abortion in the first trimester.

Factors	Adjusted OR	95% CI	*p*-Value
Maternal age (>35 years)	1.81	1.48–2.15	<0.001
Overweight status (≥25 kg/m^2^)	1.03	0.86–1.19	0.192
Smoker	1.22	1.03–1.36	0.003
Presence of chronic conditions	1.18	1.06–1.35	0.018
Previous SARS-CoV-2 infection	0.94	0.72–1.14	0.149
Abnormal uterine or cervical anatomy	1.33	1.10–1.68	<0.001
Previous miscarriage	2.02	1.54–2.61	<0.001
Assisted reproductive techniques	1.09	0.94–1.15	0.042
**Vaccine type**			
Pfizer BNT162b2	1.04	0.91–1.12	0.086
Moderna mRNA-1273 ^	1.02	0.89–1.08	0.175
**Number of doses**			
1 dose ^	0.91	0.69–1.08	0.338
2 doses	0.94	0.66–1.05	0.247
3 doses	0.77	0.60–1.01	0.590

^—reference category.

## Data Availability

The data presented in this study are available on request from the corresponding author.
